# Reading: How Readers Beget Imagining

**DOI:** 10.3389/fpsyg.2020.531682

**Published:** 2020-09-24

**Authors:** Sarah Bro Trasmundi, Stephen J. Cowley

**Affiliations:** Centre for Human Interactivity, Faculty of Humanities, University of Southern Denmark, Odense, Denmark

**Keywords:** embodied cognition, imagination, aisthesis, reading, dialogicality, distributed language, languaging, cognitive ethnography

## Abstract

We trace reading to an embodied synthetic process that drives the rapid scales of imagining. As sensorimotor engagement with written artifacts permeates experience, it sharpens the sensibility that brings forth understanding. We thus trace material engagement with written artifacts to fine control over saccadic eye movements and voicing that draws on humans or what the Greeks knew as *aisthesis*. In reading, we identify aisthesis in how prereflective judgments punctuate the flow of engagement with written documents. While the study of reading often begins with “texts,” we start with how written artifacts are put to use. We use cognitive ethnography to trace reading to how fine multiscalar coordination enables readers to engage with written artifacts such as books. Our ethnography of reading provides descriptions of how readers use sensorimotor activity to integrate understanding with saccading and actual or imagined vocalization in ways that show how reading connects sensorimotor schemata with highly skilled use of written artifacts. By pursuing the power of rapid multiscalar dynamics, we complement views of reading as slow-scale subjective experience. Rather than focus on interaction between a reader and an imagined author, we turn to coordinating with an affordance-rich environment. Human prereflective judgments demonstrably use collective experience with written signs. In fine-grained analysis of authentic data, we therefore track kinesthetic experience to how a child’s vocalizations beget understanding and, at once, imagining. These observations show how engagement brings life to written signs by connecting other peoples’ pasts with the use of gaze, gesture, voice, and touch. While describing saccades and bursts of vocalizing, we reach beyond analogies with interaction and, in so doing, the multiscalar approach takes enactive-ecological work beyond the slow interactional and social scales or reported experience. Imagining arises as readers use multiscalar happenings to bind the anticipated, the seen, and collective aspects of experience.

## Introduction: Reading Mechanisms and Imagination

As Di Paolo et al. (2018, p. 304) modestly suggest: “we are still a long distance from being able to say what happens while we are reading a text.” Likewise, Dehaene, a neuropsychologist with expertise in the reading brain, states that reading, at first sight, appears to be almost magical and a special talent that our brain was not originally designed for. According to him, a true science of reading is only recently coming into being, and it deals with broad questions such as “how is a reader able to immediately understand written marks in ways that opens up imagination?” “Why do readers take delight in reading small stories and ancient tomes?” “What are the underlying mechanisms that allow a reader to draw on a social technique of reading as well as lived experience to accomplish the amazing feat, we call reading?” And we add “what role does the living body play for imagination?”

In this paper, we thus aim at extending our grasp of reading mechanisms that allows for imagination. While imagination is prompted by material engagement with the book, its enabling conditions are traced to multiple timescales that link lived experience, norms, expectations, and anticipations. We use cognitive ethnography by turning to how readers’ engagement with books are enabled by continuous small-scale, observable judgments (hesitations, gaze, pitch) that are traced to multiple sensibilities as well as to functional sociocultural values and norms. Following Dehaene, such a multitemporal scope is necessary, as reading draws on an ontogenetic history that rewires the brain and, as we suggest, uses a primate history of engaging with artifacts. Our argument is based on an evolutionary model of human material culture that helps us understand the enigma that when we read highly sophisticated and human-made marks on paper and screens, we use “a primate brain originally designed for life in the African savanna” (Dehaene, 2009, p. 4). That the modern human is the only species with the cultural ability of sophisticated reading is a riddle that relates to the human capability to stabilize actions over time *via* language, inscriptions, and other tools that are results of human material engagement. Material engagement is thus an embodied condition for the establishment of human culture (Dehaene, 2009; Malafouris, 2013).

Even though radical embodied cognitive research is opening the domain of what happens during reading, approaches continue to lack an account of *how* reading allows for imagination (or imaginings) and understanding without making appeal to classical functionalism. Even neuroscience reaches bedrock in the attempt to explain how understanding emerges because it lacks an account of how experience and cultural norms and values impact here-and-now sensations and the local judgment that enables synthesis and opens up imagination. Further, a reader also draws on experience that cannot be described from a standard linguistic meaning-making perspective. By that, we mean that a reader has lived experience, which matters for continuous judgments of the written page. The reader’s skilled eyes and body “give life to what would otherwise remain a dead letter” and thus involves something very different from decoding letter–sound correspondences. Different from nonprimate material engagement, such as nut-cracking behavior, reading is not just functional and hedonic. Rather, we argue that it involves overall human sensibility, a set of ever more refined prereflective abilities that Montani (2017, 2019) traces to human aisthesis. Specifically, aisthesis arises as one gains experience of attending to one’s engaging with material properties of the world. As a result, people develop expertise of sensibility. In the case of reading, as shown below, it depends on timing how we saccade (and move) while drawing on expectations and feelings. In that it is prereflective, one cannot set out to explain why it happens or what it means; one can only track evidence for its occurrence. It plainly includes echoes of previous seeing, hearing, smelling, tasting, and touching. Aisthesis thus draws on continuous prereflective judgments that arise in local engagement with visible patterns on a page. The resulting ways of looking feeling and, in some cases, vocalizing are constrained by how one draws on expectations, emergent properties of the situation, the tools with which one is engaged and the historicity of the engaging body, in this case as a reader.

Embodiment is thus a necessary condition for imagination, and it opens up for an understanding of prereflective structures that comprise recurrent patterns of sensorimotor experience: we have learned to *appreciate* certain perceptions from a history with storytelling and reading that involves more than just functional judgments. While we do not know how or when nonfunctional and nonhedonic judgments arose, Montani (2019) argues that they have become important in the last 50,000 years – and, since they are learned, they must be traced to the evolution of ontogeny. Crucially, they draw on group values that we claim are central to human sensibility as appears in reading. “*There are almost no human societies that do not practice some form of drawing or engraving, be it on rock, mud or the human body. These forms were already amazingly well mastered by our ancestors in the upper Paleolithic age*” (Dehaene, 2009, p. 313). In contrast, no other species of monkey or great ape created and valued something similar that was passed on, developed and manifested through aesthetic judgments. Species such as bearded capuchins that crack nuts and seek out lizards using sticks – they too draw on material engagement to develop skills that they use to change cultural techniques. However, they do not show any signs of aesthetic judgment. Similarly, while nonhuman primates can learn to recognize symbols, they tend to use them functionally and to gain rewards. Certainly, they do not seek aesthetic outcomes or engage in moral reasoning; that is, they do not use symbols to take or change perspectives. In primates, the use of techniques is learned in ontogeny and thus integrates evolutionary, developmental, and individual timescales.

However, the multiscalarity of modern human agency reaches beyond that of other primates, in part, because humans make continuous judgments that are constrained by how ontogenetic history builds on social values and lived experience. In cognitive archeology and, above all, material engagement theory, their skills are traced to *modern* use of material artifacts. In making late stone age pottery, for example, human artifacts link individual skill with cultural style. We define such artful actions as the hallmark of aesthetic judgment. For Malafouris, the actions feature semiotic aspects that pertain to groups – showing that aisthesis draws on but is not to be explained by sociocultural organization. Judgments of skill and style thus arise as a flow of felt responding that arises in fitting actual experience with *de facto* expectations. They dominate talk-in-interaction and were originally traced to “contextualization cues” (Gumperz, 1982) or, in modern terms, by the play of intercorporeality.

For example, when South Asians offered “gravy,” their speech was often perceived as unfriendly. This aesthetic judgment was traced with a falling prominence (over around 200 ms): it was a reflective judgment or felt reaction (see, Cowley, 2006). Felt reactions occur in all modalities and can be described as interactional synchrony, accommodation, sensorimotor empathy, attunement, entrainment, and, importantly, how infant-caregiver activity comes to be coregulated. We insist that the sensibility shown – aisthesis – is irreducible to the functional and hedonic. On a third person view, the results have aesthetic/axiological elements that contribute to infant musicality (Cowley, 2003) that arises from being moved and the *intrinsic motive formation* (Trevarthen, 1999) that results from “primary intersubjectivity” (Trevarthen, 1979). While the coentrainment has been debated for 40 years – there is no doubt that *de facto* judgments influence interaction, attachments, and, importantly, how a child develops. Later in the discussion section, we use the work of Rossmanith et al. (2014) to illustrate aisthesis in the musical vocalization of “velvety soft nose” (placing prominence on the syllable in bold).

Talking draws on pico-dynamics that draw on experience, expectations, and ways of orienting to a situation (and artifacts). We claim not only that reading is a mode of action but that it also draws on aesthetic judgments that integrate histories that draw on many temporal scales. Where rendered aloud, these are enacted in vocal modulations (or prosody) that are part of flow, shape, and felt reactions. These are the hallmark of aisthesis, which, while having a “subjective” aspect, is too subtle for first-person description because the judgments are too culturally complex and far too fast to be conceptualized in real time. This view is important for the current debate of agency within ecological psychology and enactivism. No appeal to a person level of description, an organism–environment system, or autonomy can capture this multiscalar depth. In observing a reader, we emphasize that humans are strikingly heteronomous.

Further, it has often been within psychology that we find models that treat reading as individual and computational. With a recommendation from neuroscience to study the cultural, anticipatory, and experiential basis for reading, we thus challenge this view. By example, Dehaene (2009) argues that the saccading mechanisms involved in reading reflect cultural techniques that relate a cultural-dependent visual exploration strategy to a particular language and script (Dehaene, 2009, p. 17). Further, readers immediately perceive sounds, and there is abundant proof that this almost automatic process relates to our skill in linking multiple sensational experience, such as vocalizations, to what the reader saccades to. By expanding the brain-bound focus within neuroscience, we suggest that we can trace reading mechanisms to processes outside the brain, too (cf. the analysis). Specifically, we propose an ethnographic approach to observe the rapid scales of how embodied judgments are articulated on the rendering aloud. In pursuing reading, therefore, we focus on shifts in perception and felt reactions, which philosophers ascribe to the fringe of conscious experience, the proto-phenomenological and, especially, preconceptual judgments. Specifically, our concern is with the equivalent of prosody – a reader’s judgments that are neither hedonic nor, in any direct sense, functional, as we emphasize in the analysis.

Accordingly, as in the enactive-ecological tradition, we reject approaches that trace reading to the use of verbal structures (as in structural narratology or the individualistic-based approaches to text interpretation in communication studies). We fully endorse Popova’s epistemological challenge to individualistic views where narratives reflect “autonomous and self-contained worlds” (Popova, 2014, p. 322). However, we do not adopt her focus on sociointeractional relation between intentions or viewing as “expressions of intersubjective meaningful action and participatory sense-making between tellers (narrators) and readers” (p. 321). Our work contrasts with that of Popova and colleagues in that we do not ask how, in principle, stories and texts are understood over minutes and hours. Given an interest in events that bear on reportable experience, Popova presents her work as:

“social interactions, rather than sensorimotor ones, dominate certain human practices, specifically the production and reception of narratives […] while the agency of an individual is of great importance for sociality, it is acting for and through one another (interacting) that ultimately defines who we are. **Our human world is a social world and it takes place in large measure outside of our brains, in the common shared activity that is life**” (Popova, 2014, p. 315 our emphasis).

We too recognize that a “social world” unfolds outside of our brains and that reportable experience is important. It is thus no part of this paper to challenge the descriptive value of her rich account – just as we endorse literary readings that look beyond code views to pursue relevance theory. Rather, in rejecting the focus on an autonomous agent or person “level,” we turn to necessary conditions for expectations and judgments that shape, draw on, and, ultimately, ground a reader’s competence.

Our concern is ethnographic and far from offering explanation or philosophical argument; we present reading as based on primate intelligence and skill with material artifacts and finally how humans rely on prereflective judgments that draw on forms of sensibility that we ascribe to aisthesis. These arise as we use eyes, voice, and hands (and imaginings) in the scales of saccading or making/imagining rapid speech bursts (typically between 200 and 500 ms) of around five to eight syllables. Our focus on rapid activity and prereflective shifts in action/attention is intrinsic to socially derived forms of human understanding and imagining. The claim is, emphatically, not that the aesthetic somehow “causes” what is reported at a person “level”: rather, aesthetic skills and sensibility are part of what a person is such that, in Noë’s terminology, one can “do conscious experience” that has proto-phenomenological and prereflective aspects.

Our focus is not on careful reading or how, if well trained, one construed arrangements of digital artifacts as texts. We concur, in the terms of Di Paolo et al. (2018, p. 304), that we *can* choose to perceive such artifacts in terms of “material symbolic patterns” and, to the best of our ability, treat them as “products of linguistic bodies acting symbolically.” However, in presenting ethnographic work, we show that just as talk-in-interaction does not reduce to language use, textual interpretation is *one* aspect of whole-body activity – it is only partly “linguistic.” Again, we assume that, as a primate, a reader has hedonic and functional concerns that draw on a history of responsive understanding, the resulting expectations and experience of epistemic modes of action (“play”). By that we mean that, as primates, we draw on observation-based learning in discovering cultural techniques.

As material engagement, reading too depends, in part, on intrinsic motivations that are action and other-oriented, that is dialogical (Linell, 2009). For instance, infants are moved by the movements (and voices) of others to self-motivate by using a peculiar altricial-precocial pattern of infancy that emerged about 2 million years ago. Strikingly, human infants are musical and, remarkably, develop a tendency to babble in what we – and they – hear as pleasing. Such behavior makes the voice into a cultural tool that shapes a situated sense of what is appropriate, and as such, preconceptual judgments bind hedonic, functional, aesthetic, and axiological aspects. This behavior enables infants to develop interindividual ways of acting, as they orient not just to organisms/objects but to people and things. In so doing, they behave functionally, for pleasure, and draw on aisthesis. On their own, they *improve* their babbling.

Hence, we regard early ontogenesis as functioning, above all, in discovering a world of what Linell calls “interdependencies that do not reduce to outer cause–effect relations” (Linell, 2000, p. 2). Indeed, much human action relies on interdependencies between the material properties of artifacts and the manifest expectations of persons who may themselves be present or absent. In Linell’s terms, we depend on not only context that is realized but also a wide range of contextual resources – cues and hints at what is accessible and may be relevant; that is, in his terms, we rely on “apprehension of the environment” that, as we show, is irreducible to the functional and the hedonic. Acts of saying, sign-making, talking, and reading draw on criteria that are not derived from the person who acts. In this sense, we argue that appeal to a person “level” reifies a structuralist description and masks our dialogical constitution as living human beings. As we perceive and act, we bring other people’s past experiences into play, or in Bakhtin (1984) terms, we take part in *polyphony* by drawing on preconceptual judgments that, we contend, are not just functional and hedonic but at once draw on other forms of sensibility – ones that are associated with aisthesis.

## An Ethnography of the Rapid Scales of Imagining

We propose that our understanding of reading activity can be extended by using cognitive ethnography in scrutinizing embodiment with special attention to pico-dynamics. Repeated viewing generates rich ethnographic descriptions of material engagement and how mediated action is punctuated without reduction in qualities based on the individual, interaction, or the environment. In emphasizing how cultural experience lead to judgments, our work emphasizes human experience: “Ecological realism, briefly, is the view that the habitat (not the umwelt) exists independently of a given animal, that it contains meaning, and that this is the appropriate scale at which to investigate human and animal behavior […] The umwelt of an individual organism is neither “pre-given” nor a mental construction; it is enacted during the individual’s history of development and learning” (Baggs and Chemero, 2018, p. 12). By turning to video records and relying on ethnographic methods, we stress how information pertaining to a cultural ecosystem appears *for an observer* (Hutchins, 1995; Ormerod and Ball, 2017). We can track how the umwelt changes both for an individual perceiver (e.g., as wordings are construed) and how public felt reactions enable one to make judgments as one picks up “real” information. Empirically, we combine video-based cognitive ethnography (Hutchins, 1995; Steffensen, 2013; Trasmundi, 2020) with the tools of multimodal interaction analysis (Goodwin, 2018) that enable reading to be traced to embodied experience of material and a flow of judgments that bring forth imagining. We thus shift the weight from information (about and for agents) to how readers use looking and voicing to bring multiscalar experience to the material engagement that is a necessary basis for imagining. In putting phenomenological function at the fore, we stress how reading books – or other writing-based materials – generates punctuated experience as, in the now, people draw on aisthesis. Overall, ethnography enables us to describe shifts in the rapid dynamics of such activity and how judgments set off continuants (held gaze, marked changes in pitch, rhythm and tempo, use of drawl and breaks in the reading flow, grimaces, etc.). These events of typically ±500 ms are traced to how saccading co-occurs with imagining or voicing. In pursuing the special cases of reading aloud, we focus on what speech bursts (whose units are typically 250–500 ms) show of judgments of appropriacy. From those descriptions, we can identify how readers skillfully use collective constraints (i.e., alphabetic marks) to pull in repertoires of codependent structures that extend beyond the immediate situation.

**Figure 1:**
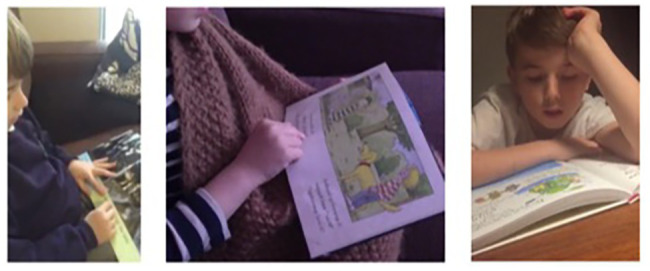
Overview of the three cases.

We explore these rapid scales in single cases from a pilot study for an ethnographic research project, *Embodied Reading*, conducted at the University of Southern Denmark^1^. The pilot project involves three ethnographic case studies collected in 2012 and 2019. The data cover aspects of a boy’s acquisition of reading skills over time. In this context, we place analytical focus on how engaging with a book brings forth imagining. The recordings took place in the boy’s home as part of a study of natural reading ecologies. In this work, we show excerpts that illustrate a variety of embodied strategies (vocalizing, saccading, and gesturing) used to bring forth imagination. ELAN software^2^ was used to annotate and transcribe video recordings. The authors (coders A and B) made the transcription and data coding individually. Specifically, the reading data use four annotation tiers: gaze, hand gestures, movements, and articulation. Reliability for observer judgments ranged from good to excellent as both coders A and B assessed the four domains of embodiments with a high degree of consistency. We suggest that, taken together, the excerpts show the multiscalar, embodied nature of reading. The data covers different stages of the boy’s reading history spanning his initial reading experiences as well as more developed skilled reading (7 years later). Observations and interviews with the boy were conducted after the video recordings were made. In the first two examples, the boy reads in English (his second language), and in the third example, his brother also participates in activity that uses Danish (his dominant language). Figure 1 above offers an overview of the cases and, in the first place, shows how bodily and nonbodily features are united in reading that is engineered in a socially organized domain or infrastructure.

## Analysis: Grounding Reading in Imagination

### Constrained Imagination: Trusting the Collective

**Figure 2:**
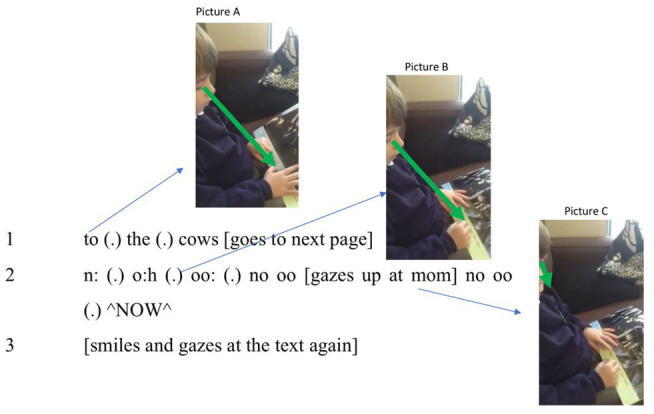
Action strategies: voice tracking, gesturing, and gazing.

In the first case, a 5-year-old boy is trying to bring learned procedures to what he sees. As one would expect, lack of experience prevents him from seeing meaningful “text.” Despite school experience with pragmatic or goal-directed strategies, he battles to render the written patterns out loud. Indeed, in zooming in on a few seconds, he is observed to switch between five embodied strategies. (1) He reads word-by-word; (2) he also reads by letter-and-syllable; (3) he looks for visible prompts in the book; (4) he uses both index fingers interchangeably (one for the left and right pages in the book respectively) to maintain attention on digits; and (5) he brings forth a more conventional talk-like burst. Given such embodied reading strategies, we show below that prereflective judgment underpins these striking shifts in ways of attending. This observation is important because those who begin with texts often appeal to “coding” and, by so doing, completely overlook, not just observables but the importance of material engagement. That is, the code metaphor deflects interest away from why and when looking and voicing are put to use. On our view, by contrast, the boy’s evolving capacity for judgment brings forth experience that rests heavily on prosody and bodily expression. The following excerpt shows the strategies in play in Figure 2.

In line 1, the boy fails to assimilate “to the cows” with his own articulatory habits by voicing syllables in staccato and not connected vocalizations. Rather than finding digital wholes, he relies on entities with quasi-phonetic or “word-like,” properties. From classroom observation, we know that this technique is favored within in the school’s cognitive ecosystem. In making use of the strategy, he shows its advantages and weaknesses. As he goes to the next page, having lost sight of the just read “cow,” seconds later, he fails to recognize “now” (line 2). Being unable to utter “now,” we will describe how he draws on aisthesis as he adopts a “letter-and-syllable” way of looking. As he changes strategy – and his way of perceiving – he uses prereflective understanding to bring forth expectations and actions as described below. First, he vocalizes [n] and seeks associations. He then treats “ow” as inviting, first [oʊ] and, when this result fails to help, he shifts to [u:] (line 2). Far from using a phonetic alphabet in *decoding*, he relies on tracking or monitoring his own vocalizations. In seeking something appropriate, he again draws on aisthesis (and his own experience). We see that as he repeats [nu:], he also seeks visible cues from mother (see picture C in Figure 2). During voice tracking, he is blocked until, suddenly, he blurts out [naʊ] in a way that is, impressionistically, triumphant. The importance of the prereflective engagement appears in his evaluation of his own empirical and perceptual actions (the multiple embodied variations of uttering n-o-w). Further, without familiarity with a language stance, he would be unable to evaluate all his variations of “now.” As noted, he utters a prosodically marked *NOW* that co-occurs with a smile and a break in the phase, which we describe as judgments that punctuate the reading flow during the creation of continuants. While, in principle, a “neural search engine” might turn up a “rule,” his strategy for shifts in vocal articulations show expertise in seeking a *suitable* way of vocalizing. After all, articulations of [aʊ] are common, and moments before, he had rendered [kaʊ] (or “cow”) out loud.

Indeed, our observations are also consistent with how “spreading activation” (Collins and Loftus, 1975) might be set off by saying “cow.” Methodologically, the observation fits the multiscalar view that extrabodily resources (and echoes of collective experience) influence human agency. The boy relies on socioculturally derived expectations that digital patterns will index familiar “words,” echoing experience with the language stance, so c-o-w is perceived or recognized *as* “the word cow.” We track that result to how his talk and gesture invite repeating, in ways that allow simple alphabets to shape phonological models. Further, as he says “now,” he uses a marked and very loud tone (^NOW^) as if announcing ownership of what he has brought forth. By focusing on vocalizing, the boy has no need for procedural knowledge (“decoding”) or interpreting (as in seeing “meaning” in the text); rather, he relies on aisthesis to trigger prereflective judgments and felt reactions. The output (^NOW^) enacts not a phonological structure but a phonetic event that resonates with positive experience (as noted, it sounds like a cry of triumph). In that way, his embodiment reveals feelings and the enactment of satisfaction at solving a problem as is displayed by a self-involving dialogical smile^3^ (line 3).

Having solved the problem, the next saccading sets off more fluent vocalizing. In what follows, we present a different strategy that, like the one above, depends on prereflective judgments or techniques of nature.

**Figure 3:**
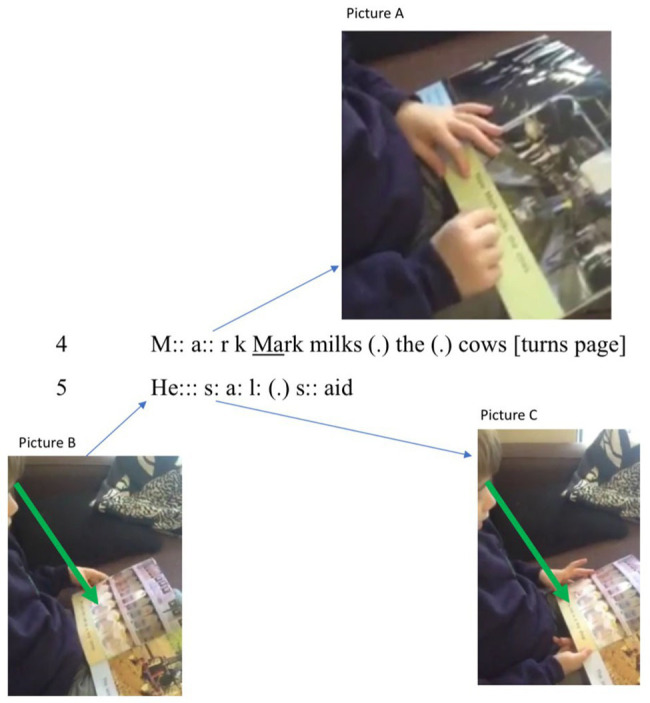
Action strategies: visual prompting, looking, and vocalizing.

First, the boy slowly sounds out [mɑ:k] (line 4) and, then, breaking with “word-by-word” or “letter-and-syllable” strategies, shifts to an unmarked burst of talk-like vocalizing: “Mark milks” (line 4). Here, the alphabetic signs become (phonetic) words that echo the digital patterns (see picture A in Figure 3). With minor hesitation, he then vocalizes, “the,” followed by a micro pause, before he utters “cows” (line 4). Fourteen seconds after his struggle with “now” in line 2, the “ow” of “cow” in line 4 prompts an actualization of an [aʊ] pattern. Then, having turned the page, he switches again. On this occasion, he uses “letter-and-syllable” as, with minimal hesitation, hearing his [sæi:] prompts him to substitute the nonphonotactic [æi:] with a standard version of “said” (viz. [sed]). Once again, we interpret this strategy as one of using the language stance in striving to fulfill expectations. As a result, he *listens to* what he vocalizes while *looking* at the page and, in this case, construing “the said” as text. Failure to glean [sæi:] “sai” seems to have triggered a negative felt reaction and, subsequently, synthesis of a familiar sound pattern (as [sed] is matched to “said”).

Appeal to decoding and text interpreta (or linguistic bodies) simply ignores a whole-bodied mix of strategies, judgments, and reliance on expectations of, in a word, the directedness of reading. Further, in ignoring the rewards of aisthesis, one fails to clarify why the boy *seeks* solutions. One simply overlooks why the boy strives to meet the standards of a wider collective and, indeed, to master fluency reading. This analysis challenges the view that *what one reads* are identified “words” or “forms” that map onto digital representations (and, for many mental or neural counterparts). Although there is no knockdown argument against appeal to the use of computational decoding rules, all such models ignore intelligent and unhidden judgments. Decoding only posits looking-based processing and not, as demonstrably occurs, activity that meets collectively defined goals. Based on the analysis, we regard the boy’s reading as aesthetic activity manifest in viable, prosodically structured wordings. The boy does not expect to be faced with nonsense, even when he only generates “quasi-words.” In short, to remain engaged, he trusts the social organization that gave digital shape to what appears on the page. As argued above, we trace this skill to expertise and making prereflective judgments as he uses the language stance and vocalizes and tracks his voice. As he does so, he creates expectations, and when they are not successful, he changes strategies and draw on how skills with the language stance prompt him to come up with possibilities. In time, he learns to articulate written marks on the paper in ways that correspond to teachers view of “text.” However, this skill, we contend, is not the basis for reading; rather, like all activity, reading has a sensorimotor basis.

**Figure 4:**
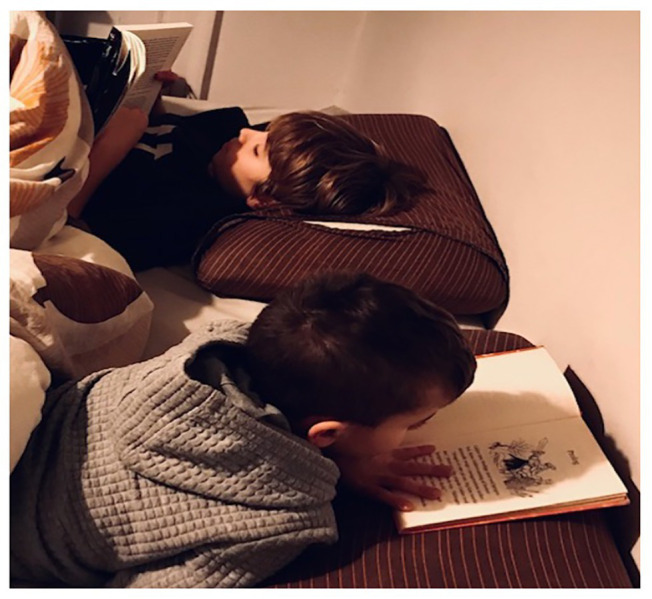
Emulating a reader.

The power of the collective is such that, at times, children trust it too much. For instance, in the case of another primary school boy – not yet a reader – he relies on emulating his older brother. In doing so, the boy organizes the reading practice, including his own position as a reader, because he can rely to only a limited extent on the social practices that are anchored in using books. This engagement is visualized below in Figure 4.

The little boy gazes in the book and, like a skilled reader, places his hands on the page to avoid the page from turning; indeed, he even synchronizes his page turning to his brother’s pace. While drawing on aesthetic judgement in attending to the page, he cannot yet perceive it *as* featuring “words.” Although the page does not afford rendering out loud (to him), he engages by pretending to read. In emulating his brother’s activity, his engagement is itself aesthetic. As argued above, tracing reading to how sensibility uses collective experiential imagination enables individual based heteronomy to draw on historical attunement and self-dialog. Even beginning readers shift strategies as they engage with marks that they are still *not able* to see as “texts.” We ascribe these shifts to prereflective promptings whose outcomes are not just functional or hedonic by arguing that they have an aesthetic basis that is, from a third person perspective, aesthetic and/or axiological. In adding value to the shifts in movement, a reader constantly monitors vocalization and, in so doing, confirms the findings of Järvilehto et al. (2011) that reading is anticipatory. Our multiscalar view of reading is crucial because a second-person orientation affords reflexivity that uses cultural norms or, in enactivist terms, new ways perceiving (Di Paolo et al., 2018). Thus, in this case, the little boy trusts and builds on already agreed descriptions and practices.

Turning to our second exemplar, we show how readers use a second-person perspective in the rapid scales of engaging with what appears on the page. Further, in anticipating understanding, the reader re-enacts experience of being interdependent with others within a given setting. As explained below, the reader’s use of a language stance permits the taking of multiple perspectives.

### Dialogical Readers: Voicing Others

**Figure 5:**
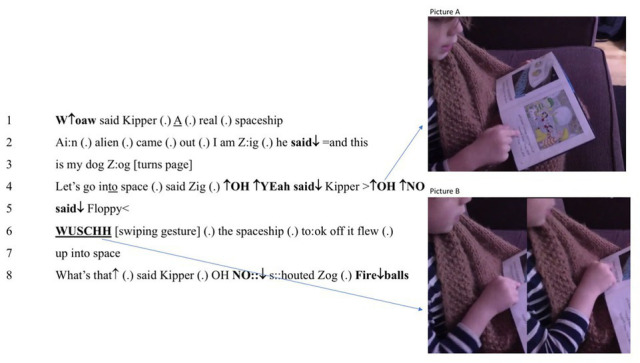
Action strategies: using only one index finger and vocalizations.

Evidence for how aisthesis shapes skills in projecting, acting, and judging the results of action are now illustrated by the same boy’s reading 2 months later. In Figure 5 below, we trace how a history of judgments like those described above have altered his action strategies. As a result, he has learned to immerse himself in the world of the book in a very different way.

Whereas the first excerpt features few “talk-like bursts” such as “Mark milks,” itself a partial repetition, this passage features many such continuants. While some are phrasal, others enact prosodically rich speech bursts of “W↑oaw, said Kipper” (line 1) or “this is my dog Z:og” (lines 2–3). Whereas a standard view invokes *looking* and phonological representation, such vocalizing shows a richness of experience that is made inexplicable by appeal to a linguistic model of “text.” Even as an early reader, the boy draws on prereflective judgment to gain prosodic control of, in these cases, connotations that echo verbal structure. Such appropriate utterances can only result from prejudgments that draw on not social or conventional construals but expert felt reactions. Further, in their connotational appropriacy, they attest to not machine-like processing but an aspiration to perform as well as others.

Here, we observe that the boy’s mix of strategies is thinning. For instance, he now relies on the left index finger only – and he no longer searches for visible prompts on the pages. Finally, we find only one case of vocalizing letter-and-syllable “Ai:n” (line 2). Indeed, his developing technical skills show that the word-by-word strategy is giving way to the use of saccade-based units. Over time, he has linked experience with rewards that draw on enhanced sensibility. Certainly, were technical, embodied skills are not part of reading, his actions (using left index finger to keep track of where he is as he vocalizes, for instance) would be hard to explain. Yet, since they aid in synthesis, we view them as part of the technique. Indeed, not only can saccading and action be synchronized, but also the boy’s vocalizations enact felt reactions. While prosody is unmarked in alphabetic writing, the boy uses it in rich ways (and not by, say, using the reading intonation of a weather forecast). Rather, as he projects connotational meanings onto what he sees as words, the boy’s judgments manifest the other-orientation of dialogicality (Linell, 2009). Rather than relying on self-involvement, he orients to moral norms and empathy. Not only does this other-orientation give the reading an aesthetic quality, but also it is manifestly axiological. Accordingly, we now sketch the phonetic shape of utterances of the ‘W↑oaw’ (line 1) and, in line 4, where he simulates Kipper’s “↑OH ↑YEah”, as well as the vocalizing of both Floppy (in line 4) and Zog’s uttering of “OH NO:: ↓” (in line 8). In these cases, too, text-based “interpretation” is powerless to explain what is observed^4^. Simply, no digital evidence bears on how prosody can or should be rendered aloud. The boy uses extratextual resources to go beyond the information given and relies on not alphabetic marks (or conventions) but linking imagined experience with felt reactions. In the case of “W↑oaw,” he finds himself prompted to use marked prosody (a rise–fall tone) that, while stereotyped, triggers a switch to a word-by-word strategy. In picking out what he sees, he makes a single use of letter and syllable to bring forth a vocalization with a curious anticipative property (in line 2). While he sees “an alien,” he overlooks the gap between the “words” by giving voice to, first, [æi:ən] and, in the saying, changing course to come up with [eɪli:ən]. He does not note the lack of “an” in rendering the wording and, in so doing, produces an utterance that chimes with common experience. This strategy shows how aisthesis enables him to evoke a collective abstraction from beyond the everyday. Far from being floored by the difficult pattern, he relies on embodied engagement with the book to exercise and extend his powers of imagination.

By using cases such as the above to build experience, he gains expertise and sensorimotor experience that, in later life, enrich prereflective judgments in orienting to written artifacts. In spite of the fact that the voice and its surrogates are usually excluded from models of reading, they seem crucial to gleaning even the unfamiliar (“alien”). The page itself acts as part of the cognitive system in that, through activity, its digital marks *become* “text for the reader.” The use of socially organized constraints allow uttering to mesh with the child’s nascent imaginary world of spaceships, aliens, and fireballs. Thus, while grounded in aesthetic experience of feeling and judgments, as we describe here, reading also enriches creative imagining. It allows for vicarious experience of emotion such as excitement, dread, etc. that pertain to not life as lived but a fictional domain. In this sense, the text brings forth *value* that is intrinsically aesthetic and inseparable from how collective domains organize culture and taste. In that sense, reading a book allows experience of dangerous emotions (as in “OH NO”) and exciting ideas while, at the same time, learning about their interrelations.

The ethnographic data confirms that the boy’s parents read aloud to him during his early years. As he has heard narratives over and over, he has rich ways of making judgments, using the voice, and in engaging with fictional universes and characters. While he can now see similarities between phrasal expressions, narrative voices in texts, etc., from the very start, reading never reduces to skilled perception of alphabetic characters (“decoding”). Rather, as in making a pot, aisthesis shapes looking, imagining (and, sometimes rendering aloud), and acting by tapping into the collective to conjure up affect and imagining. For example, in coming to feel excitement and fear in vocalizing [eɪli:ən], embodied neuropsychological models would suggest that the boy reuses neural networks (Anderson, 2014) that, later, can ground complex interpretation. As we have described, with reading, the activity is more than enactment of routines. Thus, in the second case, using a triumphant tone, the boy reads “NOW” as rhyming with “cow.” The marked prosody and concurrent smile show an expectation fulfilled. In so doing, he uses the language stance to confirm that he is right: acting in this way *is* understanding or, in another idiom, coming to act in line with a rule. Imagining is thus a constructive process that, at the best of times, gives rise to a correct outcome. In the latter examples, we see how fluency changes the activity. For example, in reading “an alien” as, first, [æi:ən] and, moments later, [eɪli:ən], the boy draws on collective imagining. He connects with a culture where [eɪli:ənz] (“aliens”) come out of spaceships. In a case such as this, he needs a language stance to treat the utterance as invoking *something* that belongs to a social domain of languaging. The peculiarities of orthography mask the phonology – ordinarily an initial “al” is pronounced /æl/unless there is a later “e” (as in “ale”). The boy’s monitoring of the failed [æi:ən], however, adds value and understanding. In the second case, multisensory activity appears in the fluency of “mark milks” and, above all, how prosody enables him to reach beyond what is said to produce vocalizations like “W↑oaw” in ways that are appropriate to the context.

In sum, our case descriptions showcase how the child does conscious experience that grants expertise and enables him to become a readerly self who is open to many perspectives. Over time, we see how he gains flexibility by using a language stance as he renders text out loud by drawing on qualities that derive from prereflective judgments.

We now focus on both more goal directed aspects of a reading process and human dialogicality as we focus on how readers bring more fully fledged imaginative powers to material engagement as they unify saccading, silent thoughts, and uttered wordings. In doing so, we open up the discussion of how, in general, reading can be traced to the aesthetic judgments that are necessary to all cases of constructive imagination. The discussion integrates other examples of reading with our ethnography of reading to emphasize that reading is based on experience of (physical) wordings and that familiarity with engaging with written artifacts allows, with experience, for the construction of fully fledged imagined worlds.

## Discussion: Imaginative Construction

So far, we have argued that, as part of radical embodied cognitive science, languaging can be traced to perpetual dialog with a collective world. On that view, readers are seen as dialogical agents. Before consolidating the argument that imagining is a constructive process, we return to the prereflective judgments that, as shown above, ground even early reading experience. In so doing, we trace the expert syntheses to felt reactions that rise as reading insinuates aesthetic and axiological dimensions into experience. Just as social coordination uses, not knowing, but sensorimotor empathy (Chemero, 2016), sensorimotor engagement enables expertise to serve in gleaning situated “meaning.” Readers use sensibility and rapid judgments as coordinated saccading prompts use of tricks and skills with inscriptions that prompt and enable them to imagine vocalizing. That means that a reader links heteronomy, including collective voices, with burgeoning experience. We have identified two fundamental ways of so doing:

- adding value to the rapid shifts in movement, and
- monitoring the results to come up with understanding.

In the case of reading, we stress that prereflective judgments engender multisyllabic bursts of vocalization. The skilled embodiment is not only more than speech production but also fits Dewey (1958) view that, in imagining, we transform possibilities into eventualities. In the case of the boy, we have described how he uses expectations based on experience with the language stance in triumphantly vocalizing “now” as akin to “cow.” The move draws on prereflective judgments and how imagining can draw on aisthesis. In what follows, we go on to expand this insight to the wider view that imagining is not an inner process but, rather, a (re)constructive mode of sensorimotor activity with general application.

**Figure 6:**
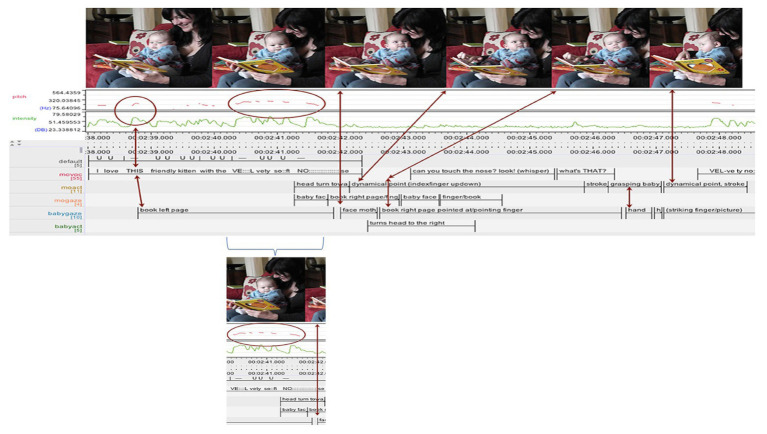
The figure is from Rossmanith et al. (2014).

As we talk or read, we use a history of social coordination that is based on linguistic embodiment that evokes, but does not reduce to, “language” or, more precisely, wordings (see, Kravchenko, 2009; Cowley, 2011; Thibault, 2011). It is by developing sensitivity to what can be (and what is) done with these physical wordings (or unique events) by performing as a skilled actor in collective, socially organized activities. As we take part in languaging, we engage in perpetual dialog with a world that amalgamates and untwines different pasts or, in Bakhtin’s terms, rely on the unfinalizability of dialog (Bakhtin, 1984). This view of wordings can be further clarified by how Rossmanith et al. (2014) in a different study describe a moment when a mother reads to an infant who, by definition, cannot understand what is said. The mother vocalizes and punctuates the flow by putting emphasis on specific syllabic patterns. Below is an overview of their example and the transcription of the reading (see Figure 6 below).

At the instant noted, the mother vocalizes “velvety soft nose.” The case exemplifies a *wording* or, for the authors, a “vocal arc.” In the case in question, it features “a gradual rise in pitch peaking in ‘VEL-vety’ followed by a slow fall in pitch and a gradual decrease in the intensity of the mother’s vocalizing, during which she turns her head toward the infant” (Rossmanith et al., 2014, p. 10). Further, the PRAAT record used shows that the wording features a marked high rising tone on *vel* (of velvety) that is far above a normal pitch range at around 300 Hz. As the authors note elsewhere, this high pitch marks “infant-directed speech” (IDS). However, this moment of IDS does not reduce to style or stereotype. At this moment, promptings set off an aesthetic match between the wordings and what is loosely called “tone.” Attention to the sonographic record shows a sprightly rhythm <VE::L:: -vi ti soft> (with similar pitch peaks and range on regularly timed syllables) that is followed by a pause. After a second, the mother and the infant move in synchrony – using, no doubt, shared rhythm – as her voice returns to the top of its range. Next, she allows her voice to fall back to a normal level as she drawls “no:::se.” In our terms, the behavioral shifts are judgments that draw on aisthesis and, almost certainly, make the utterance pleasing. Perhaps attentive readers will find it evocative of touching a tender nose. Indeed, the mother mimics just such a movement as, just 1 s later, she touches the image of a cat in the book. The case show how vocalizing can be used to beget mimetic performance. The mother links expertise with experience to make inscriptions “come alive.” The example aligns with the cases described above in showing how adult readers also rely on more than interpreting digital patterns. Finally, we suggest that this also applies to readers who rely on silent reading – and with this example in mind, this applies especially if they have experience with *the right kind of nose*. We claim that aisthesis constructs imagining in not only learners but also skilled readers that use written signs. As such, the view is relevant for all kinds of engagement with material artifacts. We now use a final brief example to spell out the point in detail.

**Figure 7:**
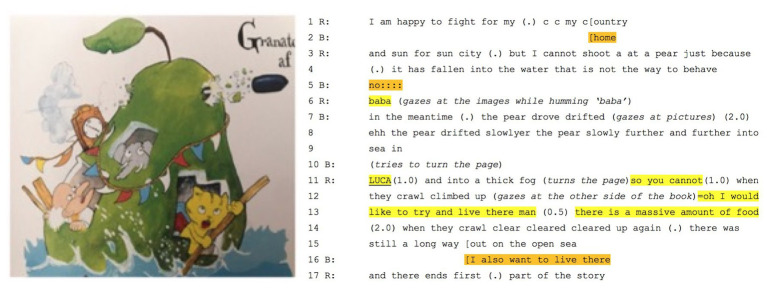
Overview of the narrative.

In returning to the boy who, now 7 years older, is reading to his little brother from *Den utrolige historie om den kæmpestore pære* (a Danish translation of *The incredible story of the giant pear*), while his younger brother has often heard the story, the boy is reading it for the first time. They are immersed in a world where an elephant, a cat, and a researcher escape from deputy major (Mr. Kvist) in a giant pear (see Figure 7 below).

**Figure 8:**
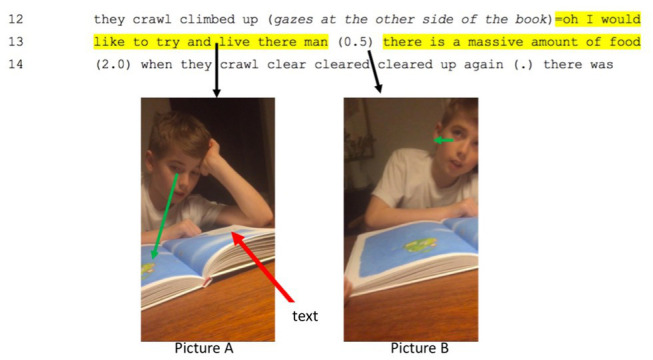
Imagining the unreal as real (red arrow indicates the location of the text field. In the second picture, R gazes at his mom, engaging with her when he imagines and explains the benefits of living in a giant pear).

As the giant pear rolls down a hill and into the sea, a colonel is instructed to shoot it with a canon. When he refuses to shoot the pear, the deputy major, Mr. Kvist, himself fires a shot that goes through its upper quarters (see the picture below). As shown in the transcription in Figure 7, in line with the marks on the page, the boy’s reading prompts him to say, first, that he would happily fight for his country and, then, that he would not shoot at a pear. As he reads on about how the drifting pear moves through a fog, he turns the page, and, in so doing, he interrupts himself (in yellow below):

His fantasy sets off a 2-s silence that would be enough to read around 20 syllables. Of course, no one can report *all* that goes on in the 2 s. However, the boy’s stance both enables his imagining of the pear – as he *says* that he would like to live there – and, as he draws on other-orientation, a shift in gaze to the picture (see picture B). As he looks, he explains: “*there is a massive amount of food*” (line 13). In arguing that imagination is synthetic and necessarily draws on aesthetic judgements, we have allowed the heard to be amalgamated with the results of gazing at the image (see picture A in Figure 8). Further, it echoes with an earlier passage where, having hollowed out the pear, the characters ate the inside. After the pause, as he finishes this part of the story, the little brother is prompted to echo his older brother’s wish (in orange in line 16 in Figure 7). Just as with case of a *velvety soft nose*, a wording evokes mimetic activity. In this case, however, the activity is not kinesthetic but, rather, linguistic. Further, the wording matters for imagination, as the little brother *also* wants to live in the pear (i.e., with his big brother – as part of an imagined world). In this case, the co-construction is dialogical in two senses: not only do the boys share a fantasy of living in a giant pear but also, importantly, they mesh with collective voices. They echo a unity of aesthetic and moral issues like serving one’s country, not shooting innocent pears and, most explicitly, the joys of inhabiting an edible fruit that can escape from cannon fire. In other words, they participate in a community. In making this claim, our work complements that of Popova and colleagues. While in dialog with the researcher, cat, and elephant, the boys are also at least metaphorically in dialog with the “author,” too (cf. Popova, 2014). Like Roald Dahl, they inhabit a collective world that “takes place in large measure outside of our brains, in the common shared activity that is life” (Popova, 2014, p. 315) and, metaphorically at least, they are in interaction with him. However, we focus on not the slow scales of reconstruction and participatory sense-making (De Jaegher and Di Paolo, 2007) but judgments that shape sensorimotor dynamics. We claim that the author and the interaction are results of a history of imagining whose grounding lies, above all, in prosody and imagined prosody based on looking and saccading. Indeed, it is because books and screens can be reconstrued that, in linguistics, emphasis falls on the verbal aspect of languaging, that is, the forms, functions, and texts of grammatical tradition. In taking a dialogical and multiscalar view, by contrast, we begin with activity and, specifically, how rapid prereflective judgments bring forth wordings (or imagined wordings) that, for many people, evoke visualizations (see, Troscianko, 2013). In all cases, these draws on sensorimotor history and, as we have argued, synthesis during which punctuated events and aisthesis beget imagining.

## Conclusion

In bringing cognitive ethnography to radical-embodied cognitive research, we use a dialogical view of language and cognition to highlight how, in reading, rapid multiscalar dynamics bring forth imagining that draws on the collective. Not only does this view support Popova’s epistemological challenge to individualistic views of narratives and “autonomous and self-contained worlds,” but our thick descriptions offer the beginnings of an account of how a reader’s experience comes to be made. Aisthesis shapes *synthesis* by tracing the technique of looking-and-vocalizing to sensorimotor engagement. Voicing and judgments depend on not the “text” but a reader who looks and brings forth wordings that amalgamate sensorimotor experience with collective use of tactile, pictorial, digital, and other expression. In other terms, neural, motor, and tactile systems bring forth this *now* and amalgamate the collective, the bodily, and experiential.

We presented fine-scaled analysis to show how reading experience is traced to punctuated bodily movement and, given its anticipatory nature, gives evidence of how readers learn to read by relying on prereflective judgments. Far from relying entirely on functional routines for dealing with “text,” the felt reactions of embodiment shape sensibility that is manifest as reading. Further, the rapid flow of punctuated events attests to the boy’s judgments or what, in Greek, was called his use of *aisthesis*. This claim is consistent with tracing aisthesis to the late stone age and changes in use of material engagement. However, in playing down conventional signs, we focus on unhidden embodied aspects of a reader’s experiential trajectory. By extending Chemero’s concept of sensorimotor empathy, we trace reading to not knowledge of language systems but expert sensorimotor experience of vocalizing. Based on the analysis, we argue that rich multiscalar events link the anticipated, the seen, and the collective in the moment of *this now*. For instance, we trace amalgamation to the activity of vocalizing and imagining, for example, “velvety soft nose.” Further, as we have argued, careful consideration can trace this multitemporality to a history of felt reactions that integrate physical wordings with expert skills in looking and vocalizing. As a result, synthetic activity – and imagining – mesh with using the words actually written (and skills based on the language stance). This view opens up new ways of describing how a reader performs as not just a person but also as a skilled participant in socially organized activity. If highly educated, a reader may even come to account for reading – and what is read – in terms of metaphorical “interactions” between her readerly self and the author of a text.

Reading thus arises because humans are partly open to and for each other. For instance, the boy draws on skills with a language stance to bring forth [naʊ] or [eɪli:ən] in ways that draw on the potential of a collective world. Readers are dialogical and other-oriented as appears in the fantasy of living in an edible pear or, indeed, fine shifts of voice and rhythm that evoke a velvety soft nose. In Dewey’s terms, material artifacts act as written signs that transform eventualities into possibilities. Empirically, we find cases where a mother is moved to mimic touching a velvety nose or when a younger brother comes to share a fantasy about living in the pear. While enabled by what linguists theorize as “text,” they manifestly use synthesis and prereflective judgments whose connotations shape mimetic behavior. Understanding thus emerges in rapid scales when people anticipate, find expectations, and fulfilled expectations (by acting as if following rules). Indeed, over time, reading skills can lead to deep resonance with what one reads, remarkable agreement on “content,” and ultimately to viewing documents as textual entities whose “meaning” appears to a reader or a critic. In our view, far from being *the basis* for reading, text serves as an ideal *result* that those who “know” the outward criteria of a given sociocultural order.

Building on the analytical results and extended discussion of the results in relation to radical embodied cognitive science, the paper makes two contributions. First, it traces a synthetic process to rapid dynamics that set off a reader’s prereflective judgments and imagining. Experience is thus enriched by engaging with artifacts and cultural memory that uses, for example, spelling systems, pets, and fantastic pears. Remarkably, the openness of human dialogicality and, inseparably, languaging transform what each of us become and what we imagine. Second, we have emphasized how imagining is traced to enskillment and expert use of sensibility that sets off aesthetic judgements that often draw on the language stance. To the extent that we are successful, we show that cognitive ethnography is a methodological tool that goes beyond first‐ and third-person views by clarifying human openness to the collective. Hardly surprisingly, imagination is like memory: it is a (re)constructive activity that blends felt reactions with others’ voices, both real and metaphorical. This appears in rapid dynamics as how people saccade, vocalize, or enact fine-scale motoric activity. Indeed, by focusing on the lived now, one brings multiscalar depth to cognitive science. Only linguistic embodiment can allow a human to imagine living in a giant pear, and we suggest that this skill requires more than picking up on affordances because one needs a dialogical agent whose felt reactions are infused with collective history. If one is to grasp reading, imagining must connect with the matters of taste that are central to the normative domains of human living.

## Data Availability Statement

The datasets generated for this study are available on request to the corresponding author.

## Ethics Statement

Ethical review and approval was not required for the study on human participants in accordance with the local legislation and institutional requirements. Written informed consent to participate in this study was provided by the participants’ legal guardian/next of kin. Written informed consent was obtained from the minor(s)’ legal guardian/next of kin for the publication of any potentially identifiable images or data included in this article.

## Author Contributions

ST designed the study and collected the data. SC and ST wrote both the theoretical framework and analysis. All authors contributed to the article and approved the submitted version.

### Conflict of Interest

The authors declare that the research was conducted in the absence of any commercial or financial relationships that could be construed as a potential conflict of interest.

## Footnotes

^1^The involved parties were informed about the data collection, protection, and data management. The participants did not receive any payment or compensation for their participation in the project. All participants and children’s parents have given consent to participate in the study, including being the subject of analysis in research publications.

^2^ELAN is a professional annotation tool developed by researchers at the Max Planck Institute for Psycholinguistics, The Language Archive, Nijmegen, the Netherlands. It is developed for the creation of complex annotations on visual data (Sloetjes and Wittenburg, 2008).

^3^Unfortunately, the smile is not possible to visualize in the picture due to poor quality, but he smiles just as he gazes down—away from the mother and back into the book.

^4^We emphasize that when arguing against text interpretation, we take interpretation to be a matter of improvising written or spoken utterances in a way that is, in some sense, equivalent to translating from text in one language to text in another.
